# Chitosan Coating with Rosemary Extract Increases Shelf Life and Reduces Water Losses from Beef

**DOI:** 10.3390/foods13091353

**Published:** 2024-04-27

**Authors:** Allison F. de Lima, Ricardo H. de L. Leite, Marília W. F. Pereira, Maria R. L. Silva, Thiago L. A. C. de Araújo, Dorgival M. de Lima Júnior, Marina de N. B. Gomes, Patrícia de O. Lima

**Affiliations:** 1 Department of Animal Sciences, Federal Rural University of the Semi-Arid, Francisco Mota Avenue, Mossoro 59625-900, RN, Brazil; henresito@hotmail.com (A.F.d.L.); marilia.filgueira@ufersa.edu.br (M.W.F.P.); raquellopes-16@hotmail.com (M.R.L.S.); thiagotor4@hotmail.com (T.L.A.C.d.A.); dorgival.junior@ufersa.edu.br (D.M.d.L.J.); pattlima@ufersa.edu.br (P.d.O.L.); 2 Department of Engineering and Technology, Federal Rural University of the Semi-Arid, Francisco Mota Avenue, Mossoro 59625-900, RN, Brazil; ricardoleite@ufersa.edu.br; 3 College of Veterinary Medicine and Animal Science, Federal University of Mato Grosso do Sul, Campo Grande 79074-460, MS, Brazil

**Keywords:** antioxidant activity, edible films, food quality, *Rosmarinus officinalis*

## Abstract

This study aimed to evaluate the influence of films based on chitosan and rosemary extract on the physicochemical, microbiological, and oxidative characteristics of beef. Refrigerated steaks of *Longissimus dorsi* were distributed in a factorial arrangement (4 × 4) into four treatments consisting of four edible films (control; chitosan; chitosan + 4% rosemary extract; and chitosan + 8% rosemary extract) and four days of aging (0, 2, 4, and 8 days). Incorporating 4% or 8% rosemary extract into the chitosan film improved the characteristics of the films in terms of moisture absorption and elasticity. The edible coatings with chitosan and rosemary extract and the different days of aging increased the tenderness and decreased the lipid oxidation of beef. In addition, the chitosan films containing rosemary extract increased the water-holding capacity and decreased the cooking losses of beef. The films containing 4% and 8% rosemary extract decreased the development of mesophilic and psychrotrophic bacteria and *Staphylococcus* ssp. in beef. We recommend incorporating 4% rosemary extract into chitosan-based coatings to preserve the quality of refrigerated beef.

## 1. Introduction

Meat is a perishable product. Its high humidity allows spoilage and pathogenic microorganisms to grow [1]. The most significant deterioration of refrigerated beef is due to microorganisms that produce undesirable organoleptic changes, unpleasant flavors, discoloration, gas, and changes in pH [2]. In this sense, refrigeration may be insufficient to keep meat on the shelf for more than 5 days. This contributes to the increase in waste of this food and impacts its marketing and food security.

Active packaging contains various active compounds, such as antioxidants; antimicrobials; and various moisture, gas, and ultraviolet radiation absorbers that interact with the packaged food or the surrounding environment [3]. Active packaging can inhibit or slow the microbiota and reactions on the food surface, where spoilage usually begins [4]. These have great potential to increase shelf life and food security [5,6]. Chitosan is among the active compounds that have recently received attention. It has been widely used in the production of coatings for various products [6,7,8].

Chitosan is an amino polysaccharide obtained from the deacetylation of chitin, one of the most abundant natural polymers in living organisms, including crustaceans, insects, and fungi. It is a non-toxic and biodegradable animal fiber [8]. The low cost of production justifies the application of chitosan because it is produced from crustacean processing waste [9]. It is characterized by a high film-forming ability, good barrier properties, biodegradability [10,11], high antioxidant activity, and substantial antimicrobial effects against a broad spectrum of bacteria [12,13]. 

Research has also focused on the incorporation of natural antioxidant additives into chitosan films to expand their use [5,6,7]. Rosemary (*Rosmarinus officinalis* L.) extract is a promising potential additive to chitosan films due to its antioxidant, antimicrobial, and medicinal properties [14,15]. Researchers have investigated the benefits of *R. officinalis* in pork, beef, and fish. They have noted the contribution of the extract to increasing the shelf life of natural meat, along with the preservation of its important nutrients. Bolumar et al. [16] found that rosemary active packaging was the best method to limit lipid oxidation induced by the high-pressure processing of pork patties. Sirocchi et al. [15] stated that packaging with rosemary essential oil and in high-oxygen conditions was the best way to prolong the shelf life of beef, extending it up to day 15. Nawaz et al. [17] concluded that chitosan and rosemary together act as an efficient preservative for fish under refrigerated storage to enhance its shelf life and prevent spoilage. We hypothesize that chitosan-based films that contain 4% and 8% rosemary extract improve the physicochemical, oxidative, and microbiological aspects of refrigerated beef. We aimed to evaluate the influence of films containing chitosan and rosemary extract on the physicochemical, microbiological, and oxidative characteristics of beef.

## 2. Materials and Methods

### 2.1. Rosemary Extract and Filmogenic Solutions

Rosemary extract was obtained according to the methodology proposed by Michiels et al. [18]. A solvent mixture (acetone/water/glacial acetic acid, 70:28:2% *v*/*v*) was used for extraction in a 1:20 ratio (i.e., 1 g of plant per 20 mL of solvent mixture).

Film-forming mixtures containing 3% chitosan and 0.6% glycerol dissolved in 2% acetic acid (*w*/*v*) were prepared. The mixtures were gelatinized for 24 h at room temperature using magnetic stirrers. Subsequently, rosemary extract was incorporated at a concentration of 4% or 8% (Table 1), under stirring for 30 min. The obtained mixtures were filtered using filter paper and autoclaved for 15 min. 

The films were produced by following the casting technique [19]. The film-forming mixture was deposited on acrylic trays and placed in an air circulation oven for 6 h at 50 °C.

### 2.2. Mechanical Properties

The breaking strength and deformation at break were determined through tensile tests on a universal testing machine (DL5000/10000 Series EMIC 23, São Paulo, Brazil). Samples (120 mm long × 25.4 mm wide) were fixed in a specific probe. The separation distance was 100 mm, and the test speed was 50 mm/s. The tests were performed in triplicate.

### 2.3. Moisture Absorption

Moisture absorption was determined according to the methodology described by Ghanbarzadeh et al. [20], in which three squares (2 cm per side) of each formulation were conditioned in a desiccator at 0% relative humidity (silica gel) for 24 h. After weighing, these were conditioned in a desiccator at 75% relative humidity (saturated NaCl solution) at room temperature for 24 h. After this second conditioning, the samples were weighed again, and the moisture absorption of the films was calculated according to Equation (1):

%ABS = [(Mf − Mi)/Mi] × 100(1)

in which %ABS is the moisture absorption percentage of the film, Mf is the final mass of the samples, and Mi is the initial mass of the samples. 

### 2.4. Thickness

The thickness of the films was determined by using a digital micrometer (Mitutoyo, 0–25 × 0.01 mm, Jundiai, São Paulo, Brazil) in five random positions near the central region of the films. The result is expressed in millimeters as the average of 10 random measurements of the film surface.

### 2.5. Solubility in Water

The soluble matter of the films was determined according to Gontard et al. [21]. Film samples (diameter = 2 cm) were immersed in 50 mL of distilled water and kept under mechanical agitation using an incubator chamber with orbital agitation for 24 h at 25 °C and 68 rpm. Subsequently, the samples were oven-dried at 105 °C for 24 h.

The soluble matter was determined with Equation (2):

DM = [(initial DM − final DM)/initial DM] × 100(2)

in which DM is the water-soluble matter (g/100 g film), initial DM is the initial dry matter of the samples (g), and final DM is the final dry matter (g) of the samples after incubation in distilled water.

### 2.6. Barrier Property

The barrier property in the ultraviolet/visible (UV/Vis) region was determined according to Fang et al. [22] using a UV/Vis spectrophotometer (Biochrom, Libra S22, Cambridge, UK). A film sample (10 cm long × 1.5 cm wide) was fixed in place in the cuvette so that the light beam passed through its surface. Transmittance was measured at wavelengths between 200 and 800 nm. Equation (3) was used:

Transparency (%) = ABS600/x(3)

in which ABS600 is the absorbance of the film at 600 nm, and x is the film thickness (mm).

### 2.7. Water Vapor Permeability (WVP)

WVP was determined according to American Society for Testing and Materials (ASTM-E96m) [23]. Circular samples (5.3 cm in diameter) were used to seal the opening of a permeation cell containing distilled water. The cell-film sets were kept in a desiccator with silica at 28 °C and 10% relative humidity.

The mass of the system was measured every hour for 12 h, and WVP was determined according to Equation (4):

WVP = Gx/t Ae P_0_ (R_1_ − R_2_) (4)

in which WVP is the water vapor permeability (g mm/h m^2^ kPa), x is the film thickness (2 mm), Ae is the exposed area (32.15 cm), P_0_ is the pressure of water vapor at 25 °C (3159 kPa), R_1_ − R_2_ is the difference in relative humidity (100), and G/t (g/h) is the linear regression angular coefficient of straight mass gain of the system versus time.

### 2.8. Color

Film color parameters—L* (luminosity (0—black, 100—white)), a* (from green (−) to red (+)), and b* (from blue (−) to yellow (+)))—were determined according to Gennadios et al. [24], using a colorimeter (HunterLab, Miniscan XE plus, Reston, VA, USA) controlled by the Universal Software (version 4.88) program. The films were superimposed on a white plate. The equipment was calibrated with black and white plates as a standard (L* = 93.9; a* = −0.8, and b* = 1.2).

### 2.9. Meat Processing and Coating Application

Beef (*Longissimus dorsi*) samples were divided into steaks of approximately 150 g (3 × 3 × 2 cm). Subsequently, they were immersed in different treatments: CO—without coating; CH—coating with chitosan; CRE4%—coating based on chitosan and 4% rosemary extract; CRE8%—coating with chitosan and 8% rosemary extract. The samples were immersed in the solutions for 1 min and then drained for 1 min. After draining, they were placed in polystyrene trays, covered with polyvinyl chloride (PVC) film, and stored at 4 °C.

The procedures were performed according to good manufacturing practices, as indicated in RDC Resolution n° 216/2004 of the National Health Surveillance Agency-ANVISA [25]. 

The steaks, with or without a coating, were evaluated at day 0 and then after 2, 4, and 8 days of storage at 4 ± 1 °C.

### 2.10. Instrumental Analyses of Coated Meat

#### 2.10.1. pH

The pH of the samples was determined using a digital pH meter (HANNA^®^ model HI 99163, Barueri, São Paulo, Brazil) coupled with a penetration electrode. The pH was measured directly in the muscle. 

#### 2.10.2. Color

Color was evaluated using a colorimeter (Konica Minolta, CM-700d/600d, Osaka, Japan). We used the CIELAB color space, of which the system considers the coordinates L* luminosity (black/white), a* red content (green/red), and b* yellow content (blue/yellow).

#### 2.10.3. Water Holding Capacity (WHC), Cooking Losses (CL), and Shear Force (SF)

The WHC was determined according to the methodology described by Hamm [26], considering the water loss released when pressure is applied to muscle tissue. The WHC was the difference between the initial and final weights, expressed as the percentage of weight lost from the initial sample.

CL were determined by weighing three portions of steak (3 × 3 × 2 cm) and then inserting them in a preheated oven at 180 °C until the temperature in the geometric center of the meat reached 71 °C. The temperature was monitored with a digital thermometer (Jprolab^®^, Paraná, Brazil). Subsequently, the samples were removed from the oven and weighed again to calculate the percentage of water loss during the thermal process.

The SF was determined from the samples that had been used to determine the CL. Three cylinders were removed per portion of cooked meat in the direction of the fibers (for a total of nine cylinders). The SF was measured with a texture analyzer (Taxt-125 Warner-Bratzler, Brookfield Engineering, Middleboro, MA, USA) [26].

#### 2.10.4. Thiobarbituric Acid Reactive Substances (TBARS)

For the TBARS test, 0.5 g of meat was mixed with the stock solution (0.375% thiobarbituric acid, 15% trichloroacetic acid, and 0.25 M HCl). Positive samples develop a pink color during heating. The absorbance of the solution was determined at 532 nm against the blank. TBARS is expressed as milligrams of malonaldehyde per kilogram of beef [27].

### 2.11. Microbiological Analysis of Coated Meat

Three meat samples from each treatment, with or without a coating, were used for microbiological analysis after 0, 2, 4, and 8 days of storage at 4 ± 1 °C. The meat samples were weighed (25 g) aseptically and transferred to sterile plastic bags, to which 225 mL of sterile buffered peptone water was added for dilutions of 10^1^ to 10^5^. 

After dilution, the samples were analyzed for the presence or absence of *Salmonella* spp. (only at day 0). The other analyses—the total count of psychrotrophic bacteria, meso-philic aerobes, and *Staphylococcus* spp.—were performed at all time points by using the official methodology for microbiological analysis to control animal products and water [28].

### 2.12. Statistical Analysis

The variables were submitted to analysis of variance by the GLM procedure in SAS OnDemand for Academics in a 4 × 4 factorial arrangement (four edible coatings—control, without coating; chitosan; chitosan plus 4% rosemary extract; and chitosan plus 8% rosemary extract) and four days of aging (0, 2, 4, and 8 days), according to the mathematical model represented by Equation (5):

Y_ijk_ = μ + S_i_ + R_j_ + S_i_ × R_j_ + ε_ijk_ (5)

in which Y_ijk_ is the dependent variable or response measured in the animal or experimental unit “k” of coatings “i” at days of aging “j”; μ is the population mean or global constant; Si is the effect of coatings “i”; R_j_ is the effect of days of aging “j”; S_i_ × R_j_ is the interaction between coating “i” and days of aging “j”; and ε_ijk_ is the unobserved random error.

The Tukey–Kramer test was used to compare the means, adopting a significance level of 5% (*p* ≤ 0.05). The same criterion was adopted to analyze the coating × days of aging interactions. 

## 3. Results and Discussion

### 3.1. Coatings

There was no difference between the films regarding breaking strength, solubility, the luminosity parameter b*, and thickness (*p* > 0.05) (Table 2).

The chitosan film had significantly different characteristics than the chitosan films with rosemary extract (*p* < 0.05) (Table 2). WVP was only different between the CRE4% and CH films (*p* = 0.04). Moisture absorption was higher in the CH film (26.56) compared with the CRE4% and CRE8% films (15.50 and 13.28, respectively; *p* = 0.0001).

WVP and water vapor absorption are important parameters for characterizing biopolymers used in designing and manufacturing edible coatings [29] and preventing food spoilage [30]. WVP has a direct relationship with thickness. Therefore, thinner chitosan films have a lower WVP [31]. Although the thickness of the films did not differ, the WVP of the films increased when 4% rosemary extract was incorporated. This result indicates a satisfactory combined effect of chitosan and rosemary extract.

Transparency was higher in the CH film and lower in the CRE8% film, while the CRE4% film had a similar transparency to both. The luminosity parameter L* differed between the CH (72.55), CRE4% (73.50), and CRE8% (69.48) films. The parameter a* was lower in the CRE8% film (1.09) compared with the CH film (1.86), while the value of the CRE4% film was similar to the CRE8% film (1.11). The decrease in parameter a* indicates a decrease in the red color intensity of the films. This may explain the reduction in this same parameter in the meat after the application of the film.

Edible films and coatings obtained from chitosan are transparent or slightly yellowish, smooth on the surface, flexible, and cohesive, with high mechanical strength (comparable to many commercial polymers), and they are hydrophilic, innocuous, biocompatible, biodegradable, and suitable for various food groups [8,32,33]. However, incorporating most extracts reduces the transparency of the film [34]. Incorporating rosemary extract also decreased the L* and a* parameters and increased the green color’s opacity and intensity. Changes in color parameters in chitosan films occur when oils or extracts are incorporated [29]. We assume that the results obtained in this study occurred due to the phenolic compounds in rosemary extract.

The incorporation of rosemary extract at different concentrations (4% and 8%) did not alter the breaking strength of the film compared with the control film and with each other. The glycerol and polysaccharide concentrations affect the stress at rupture: A high polysaccharide concentration combined with a low glycerol concentration produces films with a high breaking strength [35]. In this sense, the similarity between the treatments observed in this study can be attributed to the equal chitosan and glycerol concentrations in all treatments.

The load for deformation was higher in the CH film (14.53%) compared with the CRE4% and CRE8% films (7.70 and 8.77%, respectively; *p* = 0.0002). The mechanical properties of edible films are important to preserve their barrier behavior. Adequate mechanical strength ensures the integrity of a film and its resistance to breakage and abrasion. It reduces the occurrence of defects, such as holes or cracks, which spoil the barrier properties. Deformation at rupture measures the percentage of extension suffered by the film before its rupture, that is, the elastic capacity of the film. The decrease in deformation with the inclusion of rosemary extract may be related to a possible plasticizing effect from rosemary nanoparticles (with a diameter of 0.1–0.5 µm) well distributed within the films. They reinforce the polymer matrix [36], resulting in changes in the plasticity of the produced film.

### 3.2. Instrumental Evaluation of Coated Meat

The chitosan-based coatings, with or without rosemary extract, and the different days of aging influenced the pH; WHC; CL; SF; color parameters L*, a*, and b*; and lipid oxidation of the meat (Table 3). Moreover, there were interactions between the factors (Table 4).

#### 3.2.1. pH

We observed a gradual reduction in meat pH from uncoated meat to meat coated with CH, CRE4%, and CRE8% (*p* < 0.0001). Regarding the coating × days of aging interactions, we observed that meat coated with CRE4% or CRE8% without aging (day 0) presented a lower pH than the uncoated meat. This result indicates that rosemary extract increased the acidity of the films due to the presence of rosmarinic and carnosic acids, the biologically active constituents of the extract.

Meat loses acidity with time under refrigeration (~4 °C), becoming more susceptible to spoilage and the proliferation of pathogenic microorganisms. All coatings evaluated in this study (CH, CRE4%, and CRE8%) efficiently attenuated the increase in meat pH, with mean values lower than the uncoated meat. However, the CRE8% coating provided greater stabilization of the meat pH during aging. This can be reinforced by the coating × days of aging interactions (Table 4). The pH of the meat coated with CRE8% did not vary between days 2 (5.88), 4 (5.82), and 8 (5.88) of storage; these values are almost the same as the pH of uncoated meat at day 0 of aging (5.90). The reduction in pH during storage can be attributed to the increased concentration of organic acids (lactic and acetic acids) produced from carbohydrate metabolism by microbial enzymes [37].

Muscle pH plays a critical role in the breakdown of muscle proteins during meat storage [38]. Although there is no universal consensus, the meat’s final pH is expected to be between 5.8 and 5.4 at 24 h after slaughter. Beef with a pH above 6.0 generally denotes a quality problem, and it is less suitable for human consumption [39]. Beef with a high pH shows undesirable changes in color due to reduced oxygenation, tenderness, and water retention. In addition, meat becomes more susceptible to bacterial spoilage and has less flavor and a reduced shelf life [40]. Our results affirm that adding 4% or 8% rosemary extract to the chitosan coating efficiently maintained the ideal pH of beef stored between 0 and 8 days.

#### 3.2.2. WRC, Cl, and SF

The meat coated with CH, CRE4%, or CRE8% presented a higher WHC and lower CL compared with uncoated meat. Meat coated with CRE8% presented a higher WHC (73.57%), while we found the lowest CL for meat coated with CRE4% (26.58%) or CRE8% (27.00%). Regarding days of aging, the WHC remained higher (*p* < 0.0001) at 2, 4, and 8 days of aging, while the CL were lower when the meat was stored for 8 days compared with the other days of aging (Table 3).

Water is essential for shaping muscle structure and its consequent effects on quality. Proteins become less flexible and more rigid as water is lost from the muscle structure during heating and cooking. The rate and extent of the pH drop associated with post-mortem anaerobic muscle glycolysis are the primary determinants of the WHC of raw, processed, and cooked meat products [41]. The meat myofilaments shrink when the pH drops from 7 to 5.5 and water is expelled. Consequently, meat loses water via drip, exudate, or purge due to changes in the chemical charge and structure of proteins. Some sarcomere shortening occurs with the onset of rigor and the formation of actomyosin rigor bonds. Additionally, pre-rigor muscle temperatures below 12 °C, associated with a pH above 6, cause membrane failure, calcium release from the sarcoplasmic reticulum, and significant sarcomere shortening, thus reducing WHC. A low WHC results in high water loss from meat and meat products due to dripping and purging, representing a significant weight loss of carcasses and cuts and affecting the yield and quality of processed meat [42]. In this sense, our results affirm that chitosan coatings, especially with 4% or 8% rosemary extract, efficiently preserved the liquids in meat before and after cooking.

**Table 3:** The effects of chitosan-based coatings with and without rosemary extract and days of aging on the physicochemical parameters and oxidative stability (TBARS) of beef.

	Coating				Aging (Days)				SEM	*p*-Value		
	CO	CH	CHR4%	CHR8%	0	2	4	8		Coating	Days of Aging	C × P
pH	6.63 ^a^	6.14 ^b^	5.83 ^c^	5.77 ^d^	5.75 ^D^	6.04 ^C^	6.11 ^B^	6.47 ^A^	0.48	<0.0001	<0.0001	<0.0001
WHC	58.51 ^d^	61.12 ^c^	70.21 ^b^	73.57 ^a^	61.93 ^B^	66.87 ^A^	67.53 ^A^	67.08 ^A^	7.00	<0.0001	<0.0001	<0.0001
CL	37.52 ^a^	34.33 ^b^	26.58 ^c^	27.00 ^c^	31.90 ^AB^	31.03 ^B^	33.27 ^A^	29.23 ^C^	5.30	<0.0001	<0.0001	0.0014
Shear force	3.59 ^a^	3.03 ^b^	3.16 ^b^	2.69 ^c^	3.88 ^A^	3.27 ^B^	3.01 ^C^	2.31 ^D^	0.78	<0.0001	<0.0001	<0.0001
L*	65.51 ^c^	67.31 ^b^	68.39 ^a^	68.25 ^a^	69.58 ^A^	68.46 ^B^	67.14 ^C^	64.27 ^D^	2.97	<0.0001	<0.0001	<0.0001
a*	2.95 ^b^	3.25 ^a^	2.54 ^c^	2.51 ^c^	3.65 ^A^	2.99 ^B^	2.35 ^C^	2.26 ^C^	0.91	<0.0001	<0.0001	<0.0001
b*	8.35 ^bc^	8.17 ^c^	8.81 ^a^	8.53 ^b^	7.43 ^C^	10.67 ^A^	7.55 ^C^	8.21 ^B^	1.42	<0.0001	<0.0001	<0.0001
TBARS	0.38 ^a^	0.35 ^b^	0.21 ^c^	0.21 ^c^	0.14 ^D^	0.20 ^C^	0.40 ^B^	0.41 ^B^	0.15	<0.0001	<0.0001	<0.0001

CO = control (no coating); CH = chitosan coating; CRE4% = chitosan and 4% rosemary extract coating; CRE8% = chitosan and 8% rosemary extract coating; SEM = standard error of the mean; C × P = coating × days of aging interaction; WHC = water holding capacity; CL = cooking losses; L* = luminosity; a* = intensity of the red color; b* = intensity of the yellow color; TBARS = thiobarbituric acid reactive substances. ^abcd^ Lowercase letters indicate significant differences between the treatment means based on the Tukey test at 5% probability. ^ABCD^ Uppercase letters indicate significant differences between the time means based on the Tukey test at 5% probability.

Meat coated with CH, CRE4%, or CRE8% showed a lower SF compared with uncoated meat (*p* < 0.0001). Meat coated with CRE8% presented the lowest SF (2.69 N) (Table 3). The treatment × days of aging interactions revealed that the coatings attenuated the variations in meat texture as a function of the days of aging. This phenomenon was observed in the uncoated meat stored for 8 days, which had an inferior texture compared with the coated meat samples (Table 4).

The aging process controls and decreases muscle shortening during rigor mortis, increasing meat tenderness. During this process, enzymes act by catabolizing the tissue, increasing the fragmentation of myofibrils and contributing to meat tenderization [43]. During aging, the proteolysis of specific structural muscle proteins occurs due to the action of endogenous proteinases [44], including those activated by calcium (calpains), especially μ-calpain, and the level of its inhibitor, calpastatin, especially in the first 7–14 days post-mortem [45]. Desmin is one of the calpain substrates. It is a key structural component of muscle fiber: It connects to the Z-line and binds adjacent Z-lines [46]. In addition, differences in meat texture may be related to the coating’s WHC, keeping water inside the system and providing the juiciest and most tender meat [47]. In this sense, our results affirm that chitosan coatings, especially those containing 4% or 8% rosemary extract, efficiently preserved liquids in meat after cooking.

A plausible explanation for the observation that uncoated meat stored for 8 days presented a lower SF (1.71 N) compared with the coated samples is that, when cooled and exposed to oxidation without physical protection, the meat may suffer muscle fiber and membrane deterioration by pathogenic microorganisms, with consequent rotting. This view is consistent with our observation of the greater presence of *Staphylococcus* in uncoated meat.

**Table 4:** The effects of the interactions between the coating and days of aging on the physicochemical parameters and oxidative stability (TBARS) of beef.

Treatment	CO				CH				CRE4%				CRE8%			
Days of Aging (Days)	0	2	4	8	0	2	4	8	0	2	4	8	0	2	4	8
TBARS	0.17 ^d^	0.29 ^c^	0.49 ^b^	0.59 ^a^	0.17 ^c^	0.27 ^b^	0.47 ^a^	0.48 ^a^	0.11 ^b^	0.11 ^b^	0.31 ^a^	0.31 ^a^	0.10 ^c^	0.15 ^b^	0.32 ^a^	0.26 ^a^
pH	5.90 ^b^	6.44 ^c^	6.63 ^b^	7.57 ^a^	5.87 ^c^	6.07 ^b^	6.15 ^b^	6.45 ^a^	5.73 ^b^	5.78 ^b^	5.84 ^ab^	5.97 ^a^	5.51 ^b^	5.88 ^a^	5.82 ^a^	5.88 ^a^
WHC	57.77	60.07	57.74	58.47	55.78 ^b^	62.75 ^a^	62.96 ^a^	62.99 ^a^	63.17 ^b^	71.61 ^a^	75.02 ^a^	71.03 ^a^	71.00 ^b^	73.04 ^ab^	74.43 ^ab^	75.82 ^a^
CL	36.31 ^b^	35.31 ^b^	42.27 ^a^	36.20 ^b^	36.02	33.5	34.99	32.78	27.18 ^a^	28.16 ^a^	28.20 ^a^	22.79 ^b^	28.1	27.13	27.62	25.14
SF	4.83 ^a^	4.13 ^b^	3.69 ^b^	1.71 ^c^	3.81 ^a^	3.34 ^a^	2.56 ^b^	2.42 ^b^	3.56 ^a^	3.16 ^ab^	3.24 ^a^	2.67 ^b^	3.30 ^a^	2.47 ^b^	2.55 ^b^	2.45 ^b^
L*	70.56 ^a^	68.54 ^b^	65.46 ^c^	57.47 ^d^	69.59 ^a^	68.30 ^b^	66.81 ^c^	64.55 ^d^	69.16 ^a^	68.35 ^a^	68.60 ^a^	67.44 ^b^	69.04 ^a^	68.65 ^a^	67.71 ^b^	67.60 ^b^
a*	4.68 ^a^	3.78 ^b^	1.68 ^c^	1.67 ^c^	4.84 ^a^	3.37 ^b^	2.66 ^c^	2.13 ^d^	2.64	2.55	2.44	2.53	2.45	2.27	2.63	2.7
b*	7.56 ^c^	10.53 ^a^	6.62 ^d^	8.67 ^b^	7.40 ^b^	10.25 ^a^	7.47 ^b^	7.54 ^b^	7.38 ^c^	11.43 ^a^	7.63 ^c^	8.82 ^b^	7.37 ^c^	10.47 ^a^	8.47 ^b^	7.81 ^c^
**Days of** **Aging (Days)**	**0**				**2**				**4**				**8**			
**Treatment**	**CO**	**CH**	**CRE4%**	**CRE8%**	**CO**	**CH**	**CRE4%**	**CRE8%**	**CO**	**CH**	**CRE4%**	**CRE8%**	**CO**	**CH**	**CRE4%**	**CRE8%**
TBARS	0.17	0.17	0.11	0.10	0.29 ^A^	0.27 ^A^	0.11 ^B^	0.15 ^B^	0.49 ^A^	0.47 ^A^	0.31 ^B^	0.32 ^B^	0.59 ^A^	0.48 ^B^	0.31 ^C^	0.26 ^D^
pH	5.90 ^A^	5.87 ^AB^	5.73 ^B^	5.51 ^C^	6.44 ^A^	6.07 ^B^	5.78 ^C^	5.88 ^C^	6.63 ^A^	6.15 ^B^	5.84 ^C^	5.82 ^C^	7.57 ^A^	6.45 ^B^	5.97 ^C^	5.88 ^C^
WHC	57.77 ^C^	55.78 ^C^	63.17 ^B^	71.00 ^A^	60.07 ^B^	62.75 ^B^	71.61 ^A^	73.04 ^A^	57.74 ^C^	62.96 ^B^	75.02 ^A^	74.43 ^A^	58.47 ^D^	62.99 ^C^	71.03 ^B^	75.82 ^A^
CL	36.31 ^A^	36.02 ^A^	27.18 ^B^	28.10 ^B^	35.31 ^A^	33.50 ^A^	28.16 ^B^	27.13 ^B^	42.27 ^C^	34.99 ^B^	28.20 ^A^	27.62 ^A^	36.20 ^D^	32.78 ^C^	22.79 ^B^	25.14 ^A^
SF	4.83 ^A^	3.81 ^B^	3.56 ^B^	3.30 ^B^	4.13 ^A^	3.34 ^B^	3.16 ^B^	2.47 ^C^	3.69 ^A^	2.56 ^B^	3.24 ^A^	2.55 ^B^	1.71 ^B^	2.42 ^A^	2.67 ^A^	2.45 ^A^
L*	70.56 ^A^	69.59 ^B^	69.16 ^B^	69.04 ^B^	68.54	68.3	68.35	68.65	65.46 ^D^	66.81 ^C^	68.60 ^A^	67.71 ^B^	57.47 ^C^	64.55 ^B^	67.44 ^A^	67.60 ^A^
a*	4.68 ^A^	4.84 ^A^	2.64 ^B^	2.45 ^B^	3.78 ^A^	3.37 ^A^	2.55 ^B^	2.27 ^B^	1.68 ^B^	2.66 ^A^	2.44 ^A^	2.63 ^A^	1.67 ^B^	2.13 ^B^	2.53 ^AB^	2.70 ^A^
b*	7.56	7.4	7.38	7.37	10.53 ^B^	10.25 ^B^	11.43 ^A^	10.47 ^B^	6.62 ^C^	7.47 ^B^	7.63 ^B^	8.47 ^A^	8.67 ^A^	7.54 ^B^	8.82 ^A^	7.81 ^B^

CO = control (no coating); CH = chitosan coating; CRE4% = chitosan and 4% rosemary extract coating; CRE8% = chitosan and 8% rosemary extract coating; SEM = standard error of the mean; TBARS = thiobarbituric acid reactive substances; WHC = water holding capacity; CL = cooking losses; SF = shear force; L* = luminosity; a* = intensity of the red color; b* = intensity of the yellow color. ^abcd^ Lowercase letters indicate significant differences between the means of the days of aging within each treatment based on the Tukey test at a 5% probability. ^ABCD^ Uppercase letters indicate significant differences between the treatment means within each day of aging by the Tukey test at a 5% probability.

#### 3.2.3. Meat Color

The coatings influenced the meat color parameters, in which L* was higher in the meat coated with CRE4% or CRE8% (*p* < 0.0001). The intensity of the red color (a*) was higher in meat coated with CH, and the intensity of the yellow color (b*) was higher in the meat coated with CRE4% (Table 3). Edible coatings can alter the overall appearance of foods according to their optical properties because their color can vary depending on the type of material used for production [47]. The typical form of myoglobin associated with a low oxygen concentration (deoxymyoglobin) when the meat has an edible coating or oxygenation (oxymyoglobin) when it does not can influence the decision to purchase meat [48]. The L* and a* intensities tended to decrease as the days of aging increased (Table 3). However, the application of the coatings attenuated these effects, keeping these parameters more stable the longer the days of aging (Table 4). In the absence of oxygen, myoglobin is in the reduced form (deoxymyoglobin), which has a purple-red color. Upon exposure to air, myoglobin is oxygenated to form oxymyoglobin, which imparts a bright red color to the meat [49]. The coating slows the oxygenation process. Thus, instead of reaching the maximum value of a* after the first days of aging due to the formation of oxymyoglobin, this maximum value is reached in approximately 7 days and decreases thereafter. Our study demonstrated that changes in luminosity and coloration occurred from day 2 of aging. Our results indicate that the coatings were efficient in preserving color aspects (a*) during beef aging because the value was >10, which indicates a bright red color [50].

The a* parameter measures the redness of the meat. It is reduced at a higher pH or due to the decreased accumulation of oxymyoglobin on the muscle surface as a consequence of more intense cellular respiration [51]. The coatings with rosemary extract did not reduce the a* values when the meat was stored for 4 and 8 days, demonstrating that the coating efficiently inhibited the conversion of oxymyoglobin to metmyoglobin. These results reflect pH maintenance in the desirable range in meat stored between 4 and 8 days. Georgantelis et al. [52] found that a* values decreased as the days of aging increased when evaluating the color stability during the storage of frozen beef patties prepared with rosemary extract, chitosan, and tocopherol. They observed that beef prepared with rosemary extract and chitosan showed the smallest decrease, a finding similar to our study.

There was a variation in the b* values for all treatments from day 2 of aging. Although changes in b* values with the application of a coating are related to the thickness of the film formed on the beef [53], in our study, the incorporation of 4% or 8% rosemary extract did not influence the thickness of the film (Table 2). Our results may be more related to factors such as high meat temperature, intensity and type of light, nutrition, days of aging, and age, as described by Sañudo et al. [54].

#### 3.2.4. Lipid Oxidation

The TBARS concentration was higher in uncoated meat (0.38 mg/g malonaldehyde), followed by meat coated with CH (0.35 mg/g malonaldehyde), CRE4% (0.21 mg/g malonaldehyde), and CRE8% (0.21 mg/g malonaldehyde); the concentration was not different between the meat coated with CRE4% and CRE8% (Table 3). These results indicate that chitosan coatings enriched with rosemary extract are more efficient in maintaining meat’s oxidative stability and that 4% rosemary extract is sufficient to ensure this benefit. Oxidative processes are some of the primary mechanisms that reduce the quality of meat and meat products, with lipid, protein, and pigment degradation causing a loss of flavor, color, and nutritional value and even limiting the shelf life of these products [55]. The phenolic compounds in commercial rosemary extracts, especially carnosic acids and carnosol, are responsible for 90% of rosemary’s antioxidant properties. These act as primary antioxidants by reacting with lipid and hydroxyl radicals to transform them into stable products [56], reducing cytochrome c and removing hydroxyl radicals. These compounds can also act as metal ion chelators, thus reducing the formation of reactive oxygen species [57]. The diterpenes in rosemary extract also have significant radical-scavenging activity in both aqueous and polar media, delocalizing radical charges formed by oxidation [58]. Similarly to our study, Kahya et al. [59] studied incorporating aqueous rosemary extract into chitosan films as a potential food coating with antioxidant properties. The authors stated that the films supplemented with the extract showed a significant increase in the ability to reduce radicals estimated by the DPPH radical-scavenging assay (ranging from 10.53% to 84.46%) and the FRAP assay (ranging from 3.78 to 25.76 FeSO_4_·7H_2_O µmol eq/g dry film).

Naturally, the TBARS concentration was higher in meat that matured for a longer time due to the accumulation of peroxides. However, there was no significant difference between 4 and 8 days of aging. This can be explained by the treatment × days of aging interactions (Table 4), where the differences between the treatments (CO, CH, CRE4%, and CRE8%) were more significant as the days of aging increased (0, 2, 4, and 8 days). These responses indicate that applying a chitosan coating enriched with rosemary extract significantly impacts oxidative stability in meat undergoing aging.

#### 3.2.5. Microbiological Assessment

The different coatings and days of aging influenced the microorganism count in meat, and there were interactions between the factors (Table 5). We found the lowest microorganism counts in samples coated with chitosan and rosemary extract, with a tendency to decrease from day 4 of aging.

The coatings with rosemary extract effectively inhibited the development of mesophilic and psychrotrophic bacteria and *Staphylococcus* spp. The CRE4% and CRE8% coatings showed greater efficiency in inhibiting the development of mesophilic bacteria (6.06 and 5.93 log10 colony-forming units (CFU)/g, respectively) given that the uncoated meat and meat coated with CH showed similar results (7.15 and 7.13 log10 CFU/g, respectively). Although Brazilian legislation does not establish limits concerning mesophilic and psychrotrophic aerobic bacteria counts [60], their quantification is fundamental to assessing the deterioration of meat. Meat in which the population of mesophilic microorganisms exceeds 8 log10 CFU/g is considered deteriorated, and it presents nutritional and sensory changes [61].

**Figure 1:**
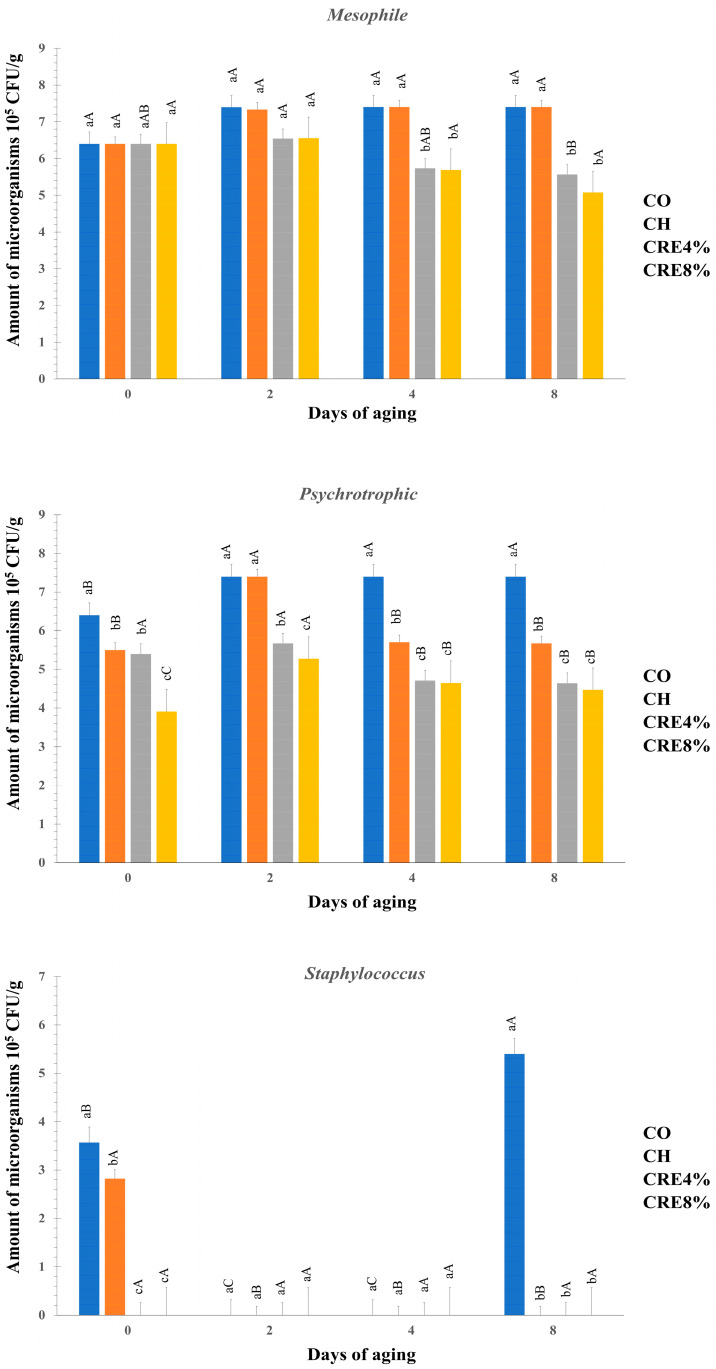
Effect of the interaction between coatings and the days of aging on the development of microorganisms in beef. The x and y axes correspond to the number of microorganisms (10^5^ colony-forming units (CFU)/g) and the aging (days). ^abc^ Lowercase letters indicate significant differences between the means of the day of aging within each treatment by the Tukey test at 5% probability. ^ABC^ Uppercase letters indicate significant differences between the treatment means within each day of aging by the Tukey test at 5% probability.

The presence of *Staphylococcus* spp. was completely inhibited at days 2, 4, and 8 of aging by the CH, CRE4%, and CRE8% coatings (Figure 1). This result indicates the immediate action of chitosan and rosemary extract on these microorganisms. This phenomenon may be attributed to the fact that Gram-positive bacteria are more sensitive to antibacterial compounds compared with Gram-negative bacteria [62]. The absence of lipopolysaccharide in the cell wall of Gram-positive bacteria might prevent the passage of active compounds through the cytoplasmic membrane [63].

*Staphylococcus aureus* is considered one of the most important causes of foodborne illness that can be transmitted through meat and meat products [64]. The suppression of its activity is influenced by packaging technology, although temperature is also a major factor [65]. The importance of pathogens like *Staphylococcus* spp. in raw foods is linked to their enterotoxigenic power, leading to gastrointestinal symptoms when ingesting contaminated food and slime and an unpleasant odor [66].

The antibacterial effect of rosemary has been demonstrated in studies with pork sausage [67], meatballs [68], boiled beef [69], and lamb meat packaged in a modified atmosphere [70]. The inhibitory effect of rosemary on the development of microorganisms results from the action of rosmarinic acid, rosmaridiphenol, carnosol, epirosmanol, carnosic acid, rosmanol, and isorosmanol. These compounds interact with the cell membrane; can alter genetic material, nutrients, electron transport, and fatty acid production; and cause the leakage of cellular components [56].

RDC Resolution n° 12 of 2001 [60] establishes only the absence of *Salmonella* spp. in a 25 g sample as a parameter of the microbiological quality of natural meat. We did not detect the presence of this pathogen in the meat analyzed in this study, demonstrating its suitability for human consumption.

## 4. Conclusions

Incorporating rosemary extract into a chitosan film improved the moisture absorption and elasticity of the films. The application of the edible coatings with chitosan and rosemary extract to beef subjected to different days of aging increased the tenderness and decreased lipid oxidation. In addition, there was an increase in the WHC and a decrease in CL. Regarding microorganism development, the coatings containing 4% or 8% rosemary extract efficiently inhibited the development of mesophilic, psychrotrophic, and *Staphylococcus* ssp. in beef. 

We recommend a chitosan-based coating with 4% rosemary extract (*Rosmarinus officinalis* L.) because it improves the preservation and quality of chilled beef, keeping it closer to natural meat. 

## Figures and Tables

**Table 1:** Composition of the film-forming matrices.

Treatment	Chitosan (%)	Glycerol (%)	Rosemary Extract (%)	Acetic Acid 2% (%)
Control (CO)	-	-	-	-
Coating 1 (CH)	3.0	0.6	-	96.4
Coating 2 (CRE4%)	3.0	0.6	4.0	92.4
Coating 3 (CRE8%)	3.0	0.6	8.0	88.4

Control = distilled water, CH = chitosan, CRE4% = chitosan and 4% rosemary extract, CRE8% = chitosan and 8% rosemary extract.

**Table 2:** Physicochemical characteristics of the films.

Item	Treatment				SEM	*p*-Value
	CO	CH	CRE4%	CRE8%		
Water vapor permeability (g mm/h m^2^ kPa)	-	0.41 ^b^	0.70 ^a^	0.59 ^ab^	0.05	0.0419
Solubility (%)	-	41.67	37.91	39.37	0.84	0.1859
Moisture absorption (%)	-	26.56 ^a^	15.50 ^b^	13.28 ^b^	2.10	0.0001
Deformation (%)	-	14.53 ^a^	7.70 ^b^	8.77 ^b^	1.09	0.0002
Breaking strength (%)	-	16.12	17.35	16.67	0.58	0.7400
Thickness (mm)	-	0.07	0.10	0.09	0.01	0.2099
Transparency (%)	-	2.56 ^a^	1.99 ^ab^	1.90 ^b^	0.13	0.0612
L*	-	72.55 ^ab^	73.50 ^a^	69.48 ^b^	0.72	0.0262
a*	-	1.86 ^a^	1.11 ^ab^	1.09 ^b^	0.16	0.0409
b*	-	32.90	27.57	29.79	1.42	0.3493

CO = control (no coating); CH = chitosan; CRE4% = chitosan and 4% rosemary extract; CRE8% = chitosan and 8% rosemary extract; SEM = standard error of the mean. ^ab^ Lowercase letters indicate significant differences between the means by the Tukey test at 5% probability.

**Table 5:** The effect of chitosan-based coatings with and without rosemary extract and the days of aging on the development of microorganisms in beef.

Microorganisms	Coating				Aging (Days)				SEM	*p*-Value		
	CO	CH	CRE4%	CRE8%	0	2	4	8		Coating	Days of Aging	C × SP
Mesophiles	7.15 ^a^	7.13 ^a^	6.06 ^b^	5.93 ^b^	6.40 ^B^	6.96 ^A^	6.56 ^AB^	6.36 ^B^	0.14	<0.0001	0.0132	0.0028
Psychrotrophic	7.15 ^a^	6.07 ^b^	5.11 ^c^	4.58 ^d^	5.30 ^C^	6.44 ^A^	5.62 ^B^	5.55 ^B^	0.20	<0.0001	<0.0001	<0.0001
*Staphylococcus*	2.24 ^a^	0.71 ^b^	0	0	1.60 ^A^	0.00	0.00	1.35 ^B^	0.29	<0.0001	<0.0001	<0.0001

CO = control (no coating); CH = chitosan coating; CRE4% = chitosan and 4% rosemary extract coating; CRE8% = chitosan and 8% rosemary extract coating; SEM = standard error of the mean; C × SP = coating × days of aging interaction. ^abcd^ Lowercase letters indicate significant differences between the means of the days of aging within each treatment by the Tukey test at a 5% probability. ^ABC^ Uppercase letters indicate significant differences between the treatment means within each day of aging by the Tukey test at a 5% probability.

## Data Availability

The original contributions presented in the study are included in the article, further inquiries can be directed to the corresponding author.
